# Exposure to *Yersinia pestis* increases resistance to plague in black rats and modulates transmission in Madagascar

**DOI:** 10.1186/s13104-018-3984-3

**Published:** 2018-12-14

**Authors:** Voahangy Andrianaivoarimanana, Minoarisoa Rajerison, Ronan Jambou

**Affiliations:** 1 Unité Peste, Institut Pasteur de Madagascar, Ambatofotsikely, P.O. Box 1274, Antananarivo, Madagascar; 2 Unité d’Immunologie, Institut Pasteur de Madagascar, Ambatofotsikely, P.O. Box 1274, Antananarivo, Madagascar; 3 Department of Parasites and Insect Vectors, Pasteur Institute, 28 rue Dr Roux, 75015 Paris, France

**Keywords:** Plague, *Rattus rattus*, F1 antigen, Madagascar, Outbreak

## Abstract

**Objectives:**

In Madagascar, plague (*Yersinia pestis* infection) is endemic in the central highlands, maintained by the couple *Rattus rattus*/flea. The rat is assumed to die shortly after infection inducing migration of the fleas. However we previously reported that black rats from endemic areas can survive the infection whereas those from non-endemic areas remained susceptible. We investigate the hypothesis that lineages of rats can acquire resistance to plague and that previous contacts with the bacteria will affect their survival, allowing maintenance of infected fleas. For this purpose, laboratory-born rats were obtained from wild black rats originating either from plague-endemic or plague-free zones, and were challenged with *Y. pestis.* Survival rate and antibody immune responses were analyzed.

**Results:**

Inoculation of low doses of *Y. pestis* greatly increase survival of rats to subsequent challenge with a lethal dose. During challenge, cytokine profiles support activation of specific immune response associated with the bacteria control. In addition, F1 rats from endemic areas exhibited higher survival rates than those from non-endemic ones, suggesting a selection of a resistant lineage. In Madagascar, these results support the role of black rat as long term reservoir of infected fleas supporting maintenance of plague transmission.

**Electronic supplementary material:**

The online version of this article (10.1186/s13104-018-3984-3) contains supplementary material, which is available to authorized users.

## Introduction

Plague, a flea-borne zoonosis, is caused by *Yersinia pestis* and is mainly associated with rodents [1]. Host resistance to plague is influenced by many factors including rodent species, genetic factors, and prior immunity. Few studies have demonstrated differences in plague resistance in wild rodents originated or not from separate field populations as reported in Western United States [2–4], in South Africa [5] and in Madagascar [6, 7]. Madagascar is the country worst affected by plague in the world with an average of 500 human cases annually especially in the central highlands [8, 9]. The black rat, *Rattus rattus*, is its main reservoir in rural areas. As reported in Antananarivo for both *R. rattus* and *Rattus norvegicus*, a higher resistance to the disease is observed in rats from endemic areas compared to those from plague-free zones [6, 7]. Genetic background, like CCR5 gene and Class I Major Histocompatibility Complex polymorphisms, seems important for this resistance l [10, 11].

Survival of *Y. pestis* within its host depends on evasion of the host’s innate immune system [12], and on circumventing phagocytosis and antimicrobial effectors [13]. In a mouse model, humoral and cellular immunity seem to be able to control the pathogen despite a down regulation of the major pro-inflammatory cytokines IFN-γ and TNF-α [14, 15]. Anti-F1 antibodies are also associated with protection against plague in vaccinated mice [16, 17]. In wild populations, co-infection with other pathogens could also play a major role in resistance to plague as during latent murine herpes virus infection. This cross protection is due to a secretion of IFN-γ along with macrophage activation in response to viral infection [18]. In addition, lipopolysaccharide produced by other pathogens during co- or previous infections may suppress *Y. pestis* virulence mechanisms by providing early recognition and initiation of immune signalling [19].

To address the questions regarding resistance in black rats, first generation (F1) of rats were obtained from wild black rats from plague-endemic and plague-free areas, and bred in the laboratory. The goals of this study were (i) to find out if pre-inoculation of *Y. pestis* can be protective following a second challenge, and (ii) to determine if genetic background influences resistance of rats to *Y. pestis*.

## Main text

### Methods

#### Establishment of F1 rat populations

F1 generations (born and bred in a laboratory) were obtained from wild *R. rattus* parents collected in the field, either from a plague endemic area (EA, Betafo) or from plague non-endemic areas (NEA, Miandrivazo and Toamasina) (Additional file 1: Fig. S1). Wild rats were kept for 2 weeks for observation and only seronegative individuals for *Y. pestis* anti-F1 IgG were selected.

#### Plague challenge experiments

A *Y. pestis* 40/09B strain isolated from bubo was used throughout this study. The bacterial concentration was estimated (i) before inoculation by measuring the optical density (OD) at 600 nm after 48 h of culture, and (ii) after injection by seeding serial dilutions of the solution on selective Cefsulodin Irgasan Novobiocin agar plates. Inoculated doses were chosen in accordance to previous results [6, 7]. Bacterial suspension (100 µl) was injected subcutaneously in the thigh. Rats were housed in a ventilated cabinet with food and water ad libitum, and were examined four times daily.

The effect of inoculation of a low dose of *Y. pestis* on response to a second lethal dose was investigated using 45 F1 rats from the EA divided into three groups (n = 15 each). Two groups were inoculated with 15 and 150 colony forming units (cfu) of *Y. pestis* respectively, whereas the control group was injected with Brain Heart Infusion (BHI). On day 29 after the first inoculation, all animals received a lethal dose of 15,000 cfu of the same strain.

Comparison of the difference in survival between F1 rats from EA and NEA parents was performed twice with two different NEAs: (i) with F1 rats originating from Miandrivazo (NEA) and Betafo (EA) (15 rats each), and (ii) a second time with F1 rats obtained from Toamasina (NEA) and Betafo (EA) (16 rats each).

To confirm that death was caused by plague, a F1 antigen diagnostic test [20] was used on spleen tissue of dead rats.

#### Detection of anti-F1 antibodies

For each experiment, plasma samples (diluted 1/100) were assayed in duplicate by ELISA for the presence of anti-F1 IgM [21] and anti-F1 IgG [22]. Results were expressed as the ratio of OD of the sample to the OD of negative sera +3 SD. Plasma samples with an OD ratio ≥ 2 were considered as positive [21].

#### Cytokine measurement

Cytokines were measured in plasma of F1 rats collected at day 5 after inoculation of 150 cfu of *Y pestis*, using a Novex Rat Cytokine Magnetic 10-Plex Panel run on a Magpix Luminex.

### Statistical analyses

Survival curves were compared by Log Rank test and at end point by Mann–Whitney test. Comparisons of percentages and means were performed using Yates’ Chi squared test and Kruskal–Wallis test respectively. Groups were compared by Mann–Whitney or Wilcoxon paired or independent test depending on the variables.

### Results

#### Effect of pre-inoculation of F1 rats with *Y. pestis* after a lethal challenge

**Fig. 1:**
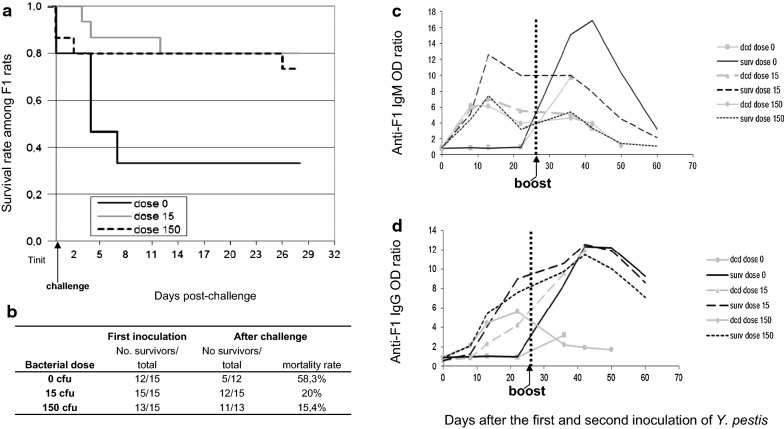
Effect of pre-inoculation of *Y. pestis* on survival of rats after challenge with a lethal dose of bacteria. Three groups of F1 rats originating from plague-endemic area were first inoculated with 0, 15 or 150 cfu of *Y. pestis* (strain 40/09B) and followed over 29 days (Tinit = time of initial inoculations). Survivors were then challenged with 15,000 cfu of the same strain and followed over 29 days. Blood samples were collected on day 0, 8, 13 and 21 after both the first and lethal inoculations of *Y. pestis*. Male/Female repartition was: 0 cfu (7/8); 15 cfu (8/7); 150 cfu (10/5). **a** Survival data is represented following Kaplan–Meier representation; **b** Data related to the curve; **c** Kinetics of anti-F1 IgM and **d** Kinetics of anti-F1 IgG antibodies from the three groups of F1 rats first inoculated with 0, 15 or 150 cfu of *Y. pestis* and challenged with 15,000 cfu are presented separately for rats surviving to the challenge (surv) and those that deceased (dcd)

After the first inoculation of *Y. pestis,* mortality was stable whatever the dose (3 rats for control group, 0 rats for 15 cfu, 2 rats for 150 cfu). After challenge with 15,000 cfu (Fig. 1a), mortality rates decreased with previous inoculation (58.3%, 20% and 15.4% mortality for 0, 15 and 150 cfu respectively, Fig. 1b). For those that died, survival time after challenge increased depending on the dose pre-injected (4.8 ± 1.4, 7.3 ± 4.9 and 15 ± 17 days for 0, 15 and 150 cfu respectively) with significantly different survival curves (*P *< 0.0364; Log Rank test). More females died compared to males after the second inoculation (17.5% vs. 12.5%), but the difference was not significant.

After the first inoculation (15 and 150 cfu) anti-F1 IgM increase between day 0 and day 8, peaked at day 13 and decreased progressively until day 22 (final mean ratio of 6.5) (Fig. 1c). Regardless of the dose injected, anti-F1 IgG appeared between day 8 and day 13 and increased up to day 22. Some surviving rats were found without anti-F1 IgG after this first inoculation (Fig. 1d).

Following the challenge, in the control group (surviving rats) a high level of anti-F1 IgM was induced (maximum at day 13 and rapid decrease before day 31 post-challenge, Fig. 1c), whereas for rats pre-inoculated with 15 cfu, IgM remained constant and declined to day 31 post-challenge. For rats previously inoculated with 150 cfu only a transitory increase in anti-F1 IgM was observed at day 7 post-challenge, rapidly declining to undetectable levels. Accordingly, anti-F1 IgG increase was faster for control group (significant increase at all days after challenge compared to day 22 after pre inoculation) than for rats pre-sensitized with 15 and 150 cfu (increase only significant at day 13 and day 31 respectively, Additional file 2: Table S1). However, more importantly for all surviving rats including controls, the same IgG pattern was observed with a maximum at day 13 (mean ratio of 12), and a slow decay until day 31 (mean ratio of 8) (Fig. 1d). These variations were significant for both IgM and IgG compared to antibodies at day 0 (P < 0.002 for all). In the same line, for surviving rats, a highly significant increase of anti-F1 IgM and IgG at day 21 after first challenge was observed compared to those that died (i.e. *P* < 0.0016, *P *< 0.0029 Mann–Whitney test).

#### Differential response of F1 rats from endemic and non-endemic areas after a single *Y. pestis* inoculation

**Fig. 2:**
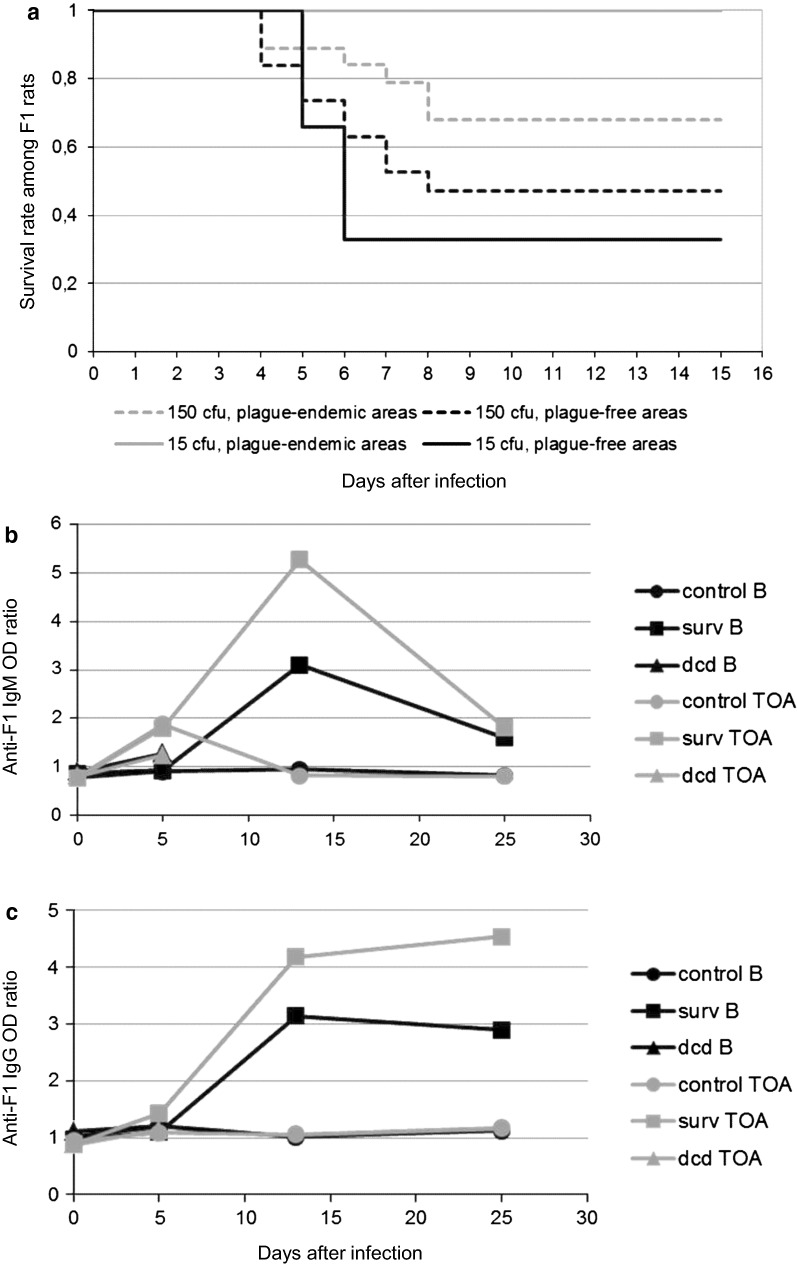
Effect of origin of rats on the survival rate following inoculation of *Y. pestis.* In the first experiment, F1 rats from plague-endemic area (Betafo) or from plague-free areas (Miandrivazo) (15 rats each) were inoculated once with 15 cfu (6 rats) or 150 cfu of *Y. pestis* (6 rats) or with BHI as a control (3 rats). In the second experiment, F1 rats from Toamasina (NEA) and Betafo (16 rats each) were inoculated once with 150 cfu of *Y. pestis* (13 rats) or with BHI as control (3 rats). Animals were blood sampled post-inoculation at day 0, 5, and 18 (Miandrivazo vs. Betafo) and 0, 5, 13 and 25 (Toamasina vs. Betafo). **a** The Kaplan–Meier survival curves are represented for each lineage: plague-endemic area (grey) and plague-free areas (black). **b** Kinetics of IgM and **c** Kinetics of anti-F1 IgG antibodies after inoculation are presented separately for surviving (surv) and deceased rats (dcd)

After single inoculation of 15 cfu, mortality was low but with a significant difference between EA and NEA (0/6 versus 4/6, *P* unilateral = 0.03). After inoculation with 150 cfu, survival rate at day 15 was higher for rats from the EA compared to others (68.4% vs. 47.3%; *P *= 0.1; Chi squared test, Fig. 2a). Most deaths occurred between day 4 and day 8 post-infection, without significant difference in the mean time to death for NEA and EA rats (5.6 ± 1.4 and 6.1 ± 1.8 days respectively).

Whatever the lineage, after single inoculation with 150 cfu, anti-F1 IgM appeared between day 5 to day 13, increased up to day 13 and then rapidly declined to a low level (Fig. 2b) without significant difference between rats (*P *= 0.38; Fisher’s exact test at day 13).

For anti-F1 IgG, a plateau was reached after day 13 (Fig. 2c), without significant difference between lineages at day 25. In the same line, for rats which died before day 8, no difference was found for anti-F1 IgM and IgG between lineages.

#### Cytokine measurement

**Fig. 3:**
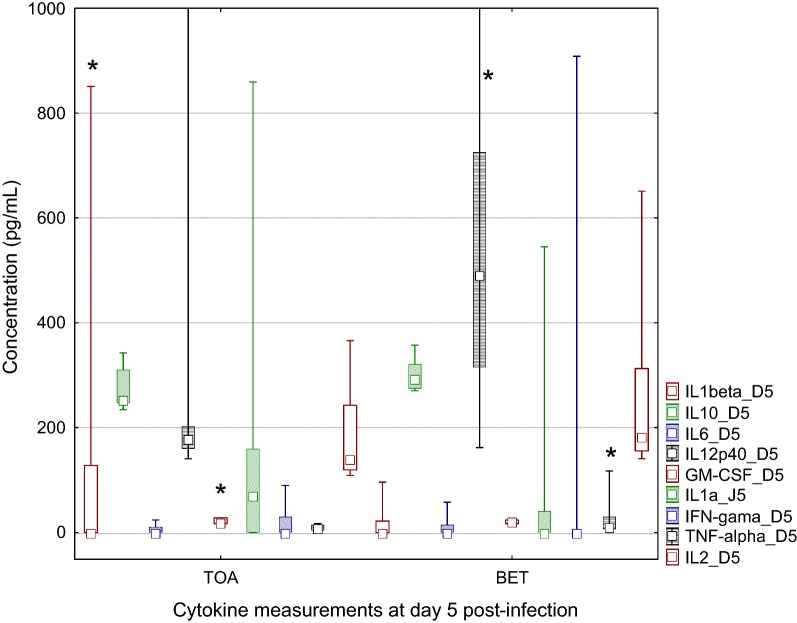
Cytokine measurements in plasma of rats after inoculation of *Y. pestis.* Cytokines measurement using a Novex Rat Cytokine Magnetic 10-Plex Panel for simultaneous quantitative determination of GM-CSF, IFN-γ, IL-1α, IL-1β, IL-2, IL-4, IL-6, IL-10, IL-12 (p40/p70) and TNF-α. Cytokine concentrations (in pg/mL) measured in plasma at day 5 post-inoculation with 150 cfu of *Y. pestis* are presented (F1 rats as in Fig. 2). Lineage Betafo and lineage Toamasina are presented separately (Plot: median/box 25–75%/min–max, *significant difference)

Cytokine measurement was obtained for eight and nine plasma samples from Toamasina (NEA) and Betafo (EA) respectively (Fig. 3). At day 0 for EA lineage, cytokine concentrations were significantly higher for IL-10, IL-6 and IL-2 (*P* < 0.07, *P* < 0.04 and *P* < 0.003 respectively), which was surprising for F1 rats housed in the same conditions. At day 5, plasma levels of IFN-γ, IL-12 were also significantly higher in rats from Betafo (EA) (*P *< 0.0001, *P *< 0.036 respectively), whereas IL-1β was higher in rats from Toamasina (NEA) lineage (*P *< 0.0001). GM-CSF significantly decreases from day 0 to day 5 in the NEA group compared to EA ones.

### Discussion

Previous studies demonstrated that rats from plague endemic area were more resistant to plague than others [6, 7] prolonging at the same time fleas survival [23]. However these local differences in resistance, could be due to co-infections by other pathogens [21]. To overcome this issue, F1-generation of rats were obtained in the laboratory from wild rats trapped in endemic and non-endemic areas.

Following inoculation of a first low dose of bacteria most of these F1 rats produced antibodies whatever the lineage. After the second lethal inoculation of *Y. pestis* both the survival rates and the time to death increased with the priming dose. It confirmed that previous contact with as few as 15 cfu of *Y. pestis* can greatly improve the survival of rats. After challenge, anti-F1 IgG antibodies increase in surviving non-inoculated rats at the same level as in pre-inoculated ones, which supports a role of IgG in the protection against *Y. pestis*. However, as previously reported with rock and ground squirrels [2] and wild rats [6, 7, 21, 24], part of them survived without any antibody production.

This study also reports a resistance of F1 rats from plague-endemic lineage compared to those from plague-free zones. This genetic hypothesis was previously evocated for rats from Antananarivo [6], in California voles [4] and in F1 progeny of grasshopper mice [3]. In grasshopper mice [4], anti-F1 titers were not linked to the origin of rats and not clearly associated with resistance. Conversely, higher concentration of IFN-γ, IL-12 and TNF-α were found in F1 rats lineage from endemic area in agreement with previous studies in susceptible mice, where down regulation of IFN-γ and TNF-α has been reported [14, 25]. Following infection of macrophages, an increase of IL-1 was also related to activation of neutrophil polynuclear cells and replication of *Y. pestis* within cells [26]. The role of TNF/TNFR1 in apoptosis related resistance to *Y. pestis* was as well recently highlighted [27]. The limited infection in resistant rats could be thus related to a lower inhibition of the innate and adaptive immune responses [28, 29], especially with a changes in phagocytic activity [4]. As for prairie dogs surviving to plague blood coagulation and inflammation may also explain this difference [30].

Overall these results confirm the potential resistance of *R. rattus* against plague and support a role of antibodies in this resistance.

### Conclusions

This study highlights two mechanisms that may support maintenance of plague in Madagascar, (i) a better heritable resistance of lineages from EA against plague and (ii) an acquired resistance against a lethal dose of *Y. pestis* initiated by pre-inoculation of a very low amount of bacteria, likely conferred by anti-F1 IgG. This suggests that rats could sustain infected flea populations and therefore making them important reservoirs of the disease [31].

## Limitations

This study was limited by a restricted number of F1 black rats obtained from wild rat lineage. More investigation are needed on a larger batch of F1 rats to address the role of genetic background in the resistance of rats from plague endemic areas.

## Additional files

**Additional file 1: Fig. S1.** Location of field collection of F1 rats’ parents. Betafo represents the plague-endemic area, whereas Toamasina and Miandrivazo are considered as plague-free areas. Dashed line: limits of the main plague-endemic area in the central highlands of Madagascar.

**Additional file 2: Table S1.** Wilcoxon paired test analysis of anti-F1 IgM and IgG variations during challenge experiment. *(pc) post challenge.

## Abbreviations

EA: endemic area
NEA: non-endemic areas
OD: optical density
Cfu: colony forming units
BHI: Brain Heart Infusion
IgG: immunoglobin G
IgM: immunoglobin M
