# Supplementary figures and images for: Exposure to Yersinia pestis increases resistance to plague in black rats and modulates transmission in Madagascar
Source: BMC Res Notes. 2018 Dec 14;11:898. doi: 10.1186/s13104-018-3984-3 (PMC6295079; doi:10.1186/s13104-018-3984-3)

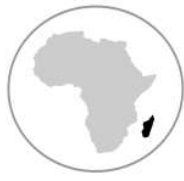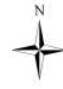

Mozambique  
Channel

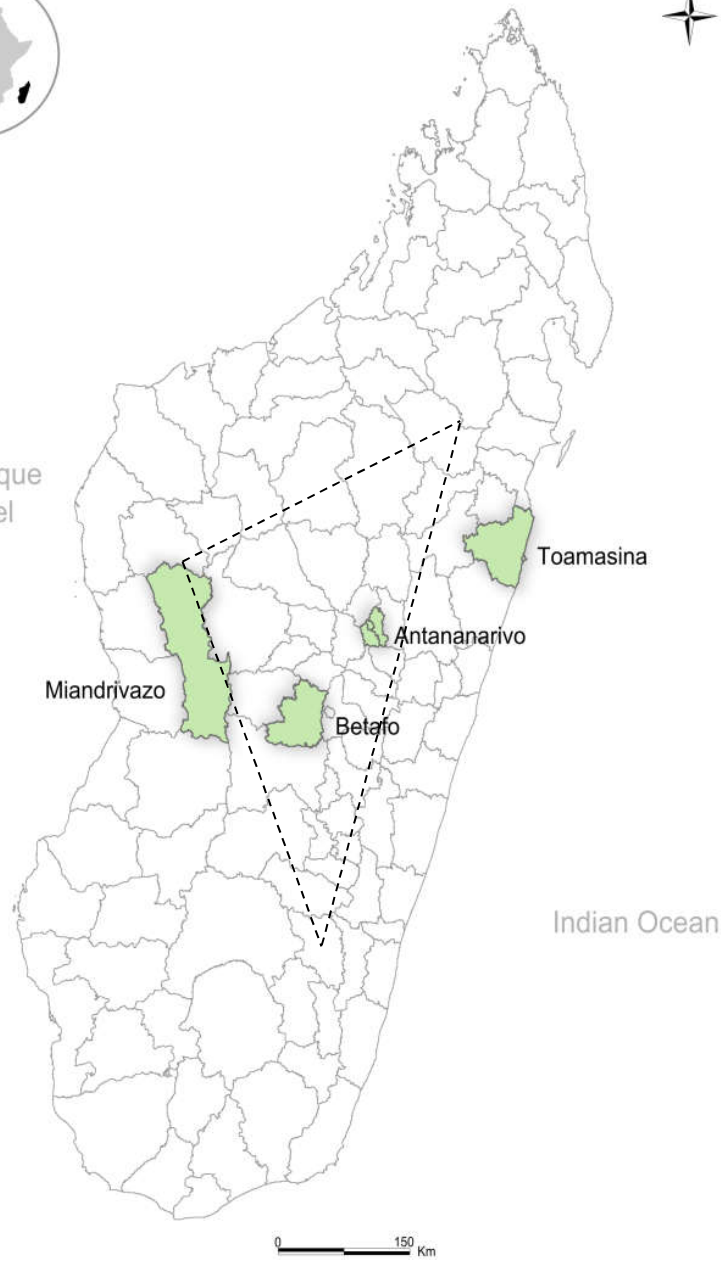

Toamasina

Antananarivo

Miandrivazo

Betafo

Indian Ocean

0 150 Km

Supplement: Supplementary file 1 — Additional file 1: Fig. S1. Location of field collection of F1 rats’ parents. Betafo represents the plague-endemic area, whereas Toamasina and Miandrivazo are considered as plague-free areas. Dashed line: limits of the main plague-endemic area in the central highlands of Madagascar. [file 13104_2018_3984_MOESM1_ESM.pdf]
