# Supplementary material for: Exposure to Yersinia pestis increases resistance to plague in black rats and modulates transmission in Madagascar
Source: BMC Res Notes. 2018 Dec 14;11:898. doi: 10.1186/s13104-018-3984-3 (PMC6295079; doi:10.1186/s13104-018-3984-3)
Supplement: Supplementary file 2 — Additional file 2: Table S1. Wilcoxon paired test analysis of anti-F1 IgM and IgG variations during challenge experiment. *(pc) post challenge. [file 13104_2018_3984_MOESM2_ESM.docx]

**Additional Table S1: Wilcoxon paired test analysis of anti-F1 IgM and IgG variations during challenge experiment**

*(pc) post challenge

|  | **First inoculation** | | | | |  | **After challenge** | | | | |
| --- | --- | --- | --- | --- | --- | --- | --- | --- | --- | --- | --- |
|  | **comparison** | **N** | **T** | **Z** | **p** |  | **comparison** | **N** | **T** | **Z** | **p** |
| Dose0 | D0_IgM & D8_IgM | 15 | 4,0 | 3,181 | 0,0015 |  | D22_IgM & D7_pc_IgM | 7 | 1,0 | 2,197 | 0,0280 |
|  | D0_IgM & D13_IgM | 15 | 3,0 | 3,237 | 0,0012 |  | D22_IgM & D13_pc_IgM | 5 | 0,0 | 2,023 | 0,0431 |
|  | D0_IgM & D22_IgM | 15 | 0,0 | 3,408 | 0,0007 |  | D22_IgM & D21_pc_IgM | 5 | 0,0 | 2,023 | 0,0431 |
|  | D0_IgG & D8_IgG | 15 | 20,0 | 2,040 | 0,0413 |  | D22_IgM & D31_pc_IgM | 5 | 0,0 | 2,023 | 0,0431 |
|  | D0_IgG & D13_IgG | 15 | 7,0 | 3,010 | 0,0026 |  | D22_IgG & D7_pc_IgG | 7 | 1,0 | 2,197 | 0,0280 |
|  | D0_IgG & D22_IgG | 15 | 35,0 | 1,420 | 0,1556 |  | D22_IgG & D13_pc_IgG | 5 | 0,0 | 2,023 | 0,0431 |
|  |  |  |  |  |  |  | D22_IgG & D21_pc_IgG | 5 | 0,0 | 2,023 | 0,0431 |
|  |  |  |  |  |  |  | D22_IgG & D31_pc_IgG | 5 | 0,0 | 2,023 | 0,0431 |
| Dose15 | D0_IgM & D8_IgM | 15 | 16,0 | 2,499 | 0,0125 |  | D22_IgM & D7_pc_IgM | 13 | 45,0 | 0,035 | 0,9721 |
|  | D0_IgM & D13_IgM | 15 | 5,0 | 3,124 | 0,0018 |  | D22_IgM & D13_pc_IgM | 13 | 44,0 | 0,105 | 0,9165 |
|  | D0_IgM & D22_IgM | 15 | 3,0 | 3,237 | 0,0012 |  | D22_IgM & D21_pc_IgM | 12 | 14,0 | 1,961 | 0,0499 |
|  | D0_IgG & D8_IgG | 15 | 6,0 | 3,067 | 0,0022 |  | D22_IgM & D31_pc_IgM | 12 | 10,0 | 2,275 | 0,0229 |
|  | D0_IgG & D13_IgG | 15 | 6,0 | 3,067 | 0,0022 |  | D22_IgG & D7_pc_IgG | 13 | 45,0 | 0,035 | 0,9721 |
|  | D0_IgG & D22_IgG | 15 | 0,0 | 3,408 | 0,0007 |  | D22_IgG & D13_pc_IgG | 13 | 0,0 | 3,180 | 0,0015 |
|  |  |  |  |  |  |  | D22_IgG & D21_pc_IgG | 12 | 9,0 | 2,353 | 0,0186 |
|  |  |  |  |  |  |  | D22_IgG & D31_pc_IgG | 12 | 32,0 | 0,549 | 0,5829 |
| Dose150 | D0_IgM & D8_IgM | 15 | 0,0 | 3,408 | 0,0007 |  | D22_IgM & D7_pc_IgM | 12 | 28,0 | 0,863 | 0,3882 |
|  | D0_IgM & D13_IgM | 14 | 0,0 | 3,296 | 0,0010 |  | D22_IgM & D13_pc_IgM | 12 | 36,0 | 0,235 | 0,8139 |
|  | D0_IgM & D22_IgM | 14 | 0,0 | 3,296 | 0,0010 |  | D22_IgM & D21_pc_IgM | 12 | 24,0 | 1,177 | 0,2393 |
|  | D0_IgG & D8_IgG | 15 | 1,0 | 3,351 | 0,0008 |  | D22_IgM & D31_pc_IgM | 11 | 7,0 | 2,312 | 0,0208 |
|  | D0_IgG & D13_IgG | 14 | 0,0 | 3,296 | 0,0010 |  | D22_IgG & D7_pc_IgG | 12 | 20,0 | 1,490 | 0,1361 |
|  | D0_IgG & D22_IgG | 14 | 0,0 | 3,296 | 0,0010 |  | D22_IgG & D13_pc_IgG | 12 | 6,0 | 2,589 | 0,0096 |
|  |  |  |  |  |  |  | D22_IgG & D21_pc_IgG | 12 | 19,0 | 1,569 | 0,1167 |
|  |  |  |  |  |  |  | D22_IgG & D31_pc_IgG | 11 | 28,0 | 0,445 | 0,6566 |
